# In Vitro Bioaccessibility of Selenium in Popular Thai Seafood Across Cooking Methods

**DOI:** 10.3390/foods15050873

**Published:** 2026-03-04

**Authors:** Narisa Rueangsri, Chonnikarn Limpaninchart, Niratchaporn Thanopajai, Kunchit Judprasong, Piyanut Sridonpai, Nunnapus Laitip, Nattikarn Ornthai, Jörg Feldmann, Alongkote Singhato

**Affiliations:** 1 Nutrition and Dietetics Division, Faculty of Allied Health Sciences, Burapha University, Chonburi 20131, Thailand; narisar@go.buu.ac.th; 2 Master of Science Program (Biomedical Sciences), Faculty of Allied Health Sciences, Burapha University, Chonburi 20131, Thailand; chonnikarn.limp@gmail.com (C.L.); niratchaporn.tn@gmail.com (N.T.); 3 Institute of Nutrition, Mahidol University, Nakhon Pathom 73170, Thailand; kunchit.jud@mahidol.ac.th (K.J.); piyanut.sri@mahidol.ac.th (P.S.); 4 Chemical Metrology and Biometry Department, National Institute of Metrology (Thailand), Pathum Thani 12120, Thailand; nunnapusl@nimt.or.th (N.L.); nattikarn@nimt.or.th (N.O.); 5 Trace Element Speciation Laboratory (TESLA), Institute of Chemistry, University of Graz, 8010 Graz, Austria; joerg.feldmann@uni-graz.at

**Keywords:** selenium, in vitro, bioaccessibility, seafood, marine foods, equilibrium dialyzability

## Abstract

Selenium (Se) is a vital element for human health and seafood represents one of its major dietary sources. Nevertheless, information regarding the bioaccessibility of Se from seafood commonly consumed in Thailand remains scarce. To address this limitation, the present study evaluated in vitro Se bioaccessibility using the equilibrium dialyzability approach. Ten seafood species frequently selected by Thai consumers were investigated to determine total Se concentrations following different culinary treatments, namely fresh, boiling, frying, and grilling. For thermally processed samples, gastrointestinal digestion was simulated in vitro through enzymatic digestion prior to bioaccessibility assessment using the equilibrium dialyzability method. Inductively coupled plasma triple quadrupole mass spectrometry (ICP-QQQ-MS) was used to precisely quantify the total and dialyzable Se fractions. The effects of seafood species and cooking methods were evaluated statistically using two-way analysis of variance and the Tukey’s honestly significant difference (HSD) test for post hoc comparisons. The findings showed that, across the majority of cooking techniques, Indo-Pacific horseshoe crab eggs consistently showed considerably greater Se contents than other seafood (*p* < 0.05). Moreover, Se bioaccessibility in Indo-Pacific horseshoe crab eggs (81.1–88.3%) was markedly greater than that observed in other seafood items, including musk crab, blue crab, oysters, and wedge shell, regardless of cooking method (*p* < 0.05). No statistically significant differences in Se bioaccessibility were observed among boiling, frying, and grilling (*p* > 0.05), indicating that thermal processing did not adversely affect Se availability. Overall, the seafood species examined in this study, irrespective of preparation method, contained substantial Se levels with high bioaccessibility, underscoring their nutritional value and supporting dietary recommendations that promote balanced consumption of marine foods.

## 1. Introduction

Since selenium (Se) is an essential trace element that contributes to immunological support, antioxidant activity, and other processes, it is well recognized that sustaining optimal health functions in the human body requires enough intake of Se [1,2]. Serum Se below 63 µg/L is considered Se deficiency [3], while the suggested daily Se intake for adults from global and Thai recommendations is 55 µg/day [4]. However, Se deficiency has been reported in many countries. For example, 13.9% of Spanish youths were found to have serum Se levels less than 60 µg/L [5], 41% of Saudi adults were reported to have Se concentrations in toenails less than 0.56 µg/g [6], and 56% of children living with HIV in Thailand were found to have low serum Se [7]. Insufficient long-term Se intake has been reported to contribute to the development of several complications. Previous studies have found that it is related to reduced immune system quality, tumor formation, and diabetes mellitus [8]. Therefore, encouraging people to consume food sources of Se is necessary to maintain their well-being.

Seafood (i.e., shrimp, crabs, shellfish, marine fish, etc.) is widely consumed worldwide and is well known as a source of high biological value protein, nutrient-dense food, and significant dietary source of Se. Previous reports have revealed Se concentrations in marine fish and other seafood species. For example, a study from Norway reported Se concentrations of 1.4 mg/kg in Wahoo and 0.8 mg/kg in flying fish [9]. In the United States, the average Se concentration in marine fish was established to range from 0.18 to 0.58 mg/kg [10]. According to a Japanese study, the level of Se in alfonsino meat was 1.27 mg/kg, but the levels in other seafood species, including squid and shellfish, varied between 0.12 and 1.20 mg/kg [11]. Another study in Mexico found that commercial shrimp contained Se concentrations ranging between 0.02 and 3.8 mg/kg [12]. In Thailand, one of the largest seafood-producing countries, a previous finding established that Se concentrations in seafood such as crabs, shrimp, and squid across various cooking methods ranged between 0.073 and 1.04 mg/kg [13].

Bioaccessibility is the fraction released from the matrix into the GI lumen (in vitro), whereas bioavailability is the fraction absorbed/systemic (in vivo) which becomes available for metabolic use or storage after absorption, distribution, metabolism, and excretion. Hence, bioaccessibility refers to the proportion of an element or compound liberated from the food matrices into the intestinal lumen via simulated gastrointestinal digestion (in vitro), quantified by its dialyzability. It mimics conditions such as enzymatic digestion, temperature, and pH to assess nutrient interactions and the nutrients released from food samples after in vitro digestion [14]. Hence, foods that are high in bioaccessibility of specific nutrients tend to be more easily absorbed, have higher bioavailability in circulation, and are efficiently utilized in human biological functions [15]. A good understanding of nutrient and trace element bioaccessibility in various foods is advantageous for promoting appropriate food choices that provide optimal nutritional benefits, especially for individuals at risk of nutrient deficiencies. Previous studies have applied this method to determine nutrient bioaccessibility, such as minerals in vegetables [16] and iron in egg [17]. For Se, due to many analytical interferences, data on Se bioaccessibility from foods remain very limited. A previous study from Spain found that the in vitro bioaccessibility of Se was relatively high, with values of 50% in tuna, 76% in swordfish, and 83% in sardines [18]. In Thailand, the only previous study using an in vitro equilibrium dialysis method reported that Se bioaccessibility from freshwater and marine fish ranged between 48.8% and 64.6%, and the study assessed only two cooking methods: boiling and frying [19].

While total Se concentrations and true retention of Se in the seafood species in Thailand were previously reported [13], the present study aimed to provide a newly generated dataset on Se bioaccessibility using an in vitro equilibrium dialysis technique to better recognize Se bioaccessibility in other popular consumed seafood species in Thailand, prepared using various cooking methods. Inductively coupled plasma triple quadrupole mass spectrometry (ICP-QQQ-MS) was used for Se determination in this study in order to reduce analytical interferences while achieving a very low limit of detection.

## 2. Materials and Methods

### 2.1. Chemicals and Reagents

Most chemicals and reagents used in this study were described in the previous study (i.e., HNO_3_, certified reference materials, standard solutions, etc.) [13]. Additional reagents used for in vitro protocols were EDTA-Ca-Na_2_, porcine bile extract (B-6831), pepsin derived from porcine gastric mucosa (P-7000), and pancreatin from porcine pancreas (P-1750), and were purchased from Sigma-Aldrich (St. Louis, MO, USA). Merck (KGaA, Darmstadt, Germany) supplied the potassium hydroxide (KOH, 105033), sodium bicarbonate (NaHCO_3_), and dialysis tubing with an internal diameter of 13 mm and a molecular weight cut-off of 12–14 kDa, whereas flat dialysis membranes (MWCO 12–14 kDa, width 15.9 mm) were purchased from Medicell Membranes Ltd. (Greenwich, London, UK). The Milli-Q^®^ purification system (Millipore Sigma, St. Louis, MO, USA) was the source of deionized water used throughout for all experimental procedures.

### 2.2. Seafood Sample Preparation

Based on information from the Thai food composition database [20] and national aquaculture statistics [21,22], ten seafood species that are extensively produced and often sold in markets across Thailand were selected. In addition, ethical approval was not required for this study because the samples consisted of commercially available seafood purchased from local markets. All purchased seafood had already been slaughtered by vendors for sale. After purchase, the samples were packed in clean plastic bags and kept on sanitary ice during transportation and handling, ensuring compliance with food safety practices and minimizing the risk of cross-contamination.

Supplementary Table S1 lists these selected species’ scientific, common, and Thai names. Approximately 500 g of each sample was randomly selected from 3–4 shops at 3 local seafood markets (Ang Sila market, Hard Won market, and Nongmon market) in Chonburi, Thailand. At Burapha University’s Nutrition and Dietetics Division, each seafood item was prepared and cooked in compliance with previously described protocols [23].

In summary, all seafood samples were recorded in grams prior to and following the removal of waste and inedible portions. Boiling was performed using deionized water (Milli-Q^®^ EQ 7000, Merck KGaA, Darmstadt, Germany); each sample meat was boiled at 100 °C for 20–30 min. Palm oil was used for frying; each sample meat was fried at 170–210 °C for 5–10 min. An electric grill pan was used for grilling; each sample meat was grilled at 68–70 °C (internal sample temperature) for 5–10 min with flipping every 2 min. After cooking, each cooked sample was homogenized using a food blender (Philips HR3760/01, Amsterdam, Netherlands) and subsequently freeze-dried at −55 °C under a pressure of 0.040 mbar for 24 h using a freeze dryer (Kinetic Engineering Co., Ltd., Bangkok, Thailand). The dried samples were transferred to screw-cap plastic containers, finely milled using a laboratory grinder (A11 Basic Analytical Mill, IKA, Staufen, Germany), and kept at −40 °C until further analysis.

### 2.3. Total Se Content Analysis in Seafood Samples

The National Institute of Metrology (Thailand) has provided the instrument, ICP-QQQ-MS (Agilent 8800 triple quadrupole ICP-MS, Agilent Technologies, Santa Clara, CA, USA), to determine the total Se contents in all seafood samples with all described cooking methods. To minimize the effect of interference, the collision and reaction gas modes were applied to enhance Se sensitivity and accuracy during operation. The standard solution of Se in 2% *v*/*v* HNO_3_ was used to generate the external calibration curve, whereas Rh in 2% *v*/*v* HNO_3_ was used as the internal standard, which was added to the digested sample solutions at the same concentration to track any changes in the ICP-QQQ-MS condition during operation.

A previous study’s procedures for creating the final solution and microwave-digesting the seafood samples were adjusted [24]. In summary, each 0.5 g of sample and CRMs (0.25 g) was weighed before 5 mL of concentrate HNO_3_ (65% *v*/*v*) was added to a glass vial. All of the samples were then microwave-assist digested in triplicate using the Anton Paar Multiwave 7000 (Anton Paar, Graz, Austria) at the settings listed in Supplementary Table S2. After that, each digested sample was weighed and brought to volume in a separate screw cap tube (50 mL) using DI water. Then, 10 mL of Rh was added as an internal standard in all prepared tubes before ICP-QQQ-MS injection (triplicate analyzed for each sample). Blank samples (digested 65% HNO_3_) were also prepared with the same described protocol to avoid sample contamination during analysis.

### 2.4. Percentage of Yield Factor Determination

Each seafood sample’s mass changes as a result of cooking was measured by the mass yield factor (YF), which takes into account the growth or loss of fat and/or water. It was computed by dividing each seafood sample’s cooked mass (g) by its raw mass (g).

### 2.5. Percentage of True Retention of Se in Seafoods Using Several Cooking Methods

An analytical scale was used to weigh each sample precisely to three significant digits, and measurements were made both before and after the cooking process to assess how each cooking technique affected the seafood samples’ ability to retain Se. The true retention (TR) of Se in the cooked samples was determined by the following formula:

$$\%\mathrm{TR}\;=\;\frac{µg\;Se\;per\;100\;g\;of\;cooked\;seafood\;\times \;mass\;of\;cooked\;seafood}{µg\;Se\;per\;100\;g\;of\;raw\;seafood\;\times \;mass\;of\;raw\;seafood}\;\times \;100$$

### 2.6. Determination of In Vitro Bioaccessibility of Se from Seafood Samples

Cooked seafood is a common feature of traditional Thai seafood cuisine and recipes; eating the selected seafood species uncooked is quite rare and not recommended due to the risk of foodborne disease [25]. Therefore, in this study, the equilibrium dialysis approach was used to study boiled, fried, and grilled seafood using the gastrointestinal simulation methodology [26]. Brief details on the sample-to-liquid ratio, target and final pH values, volumes inside and outside the dialysis bags, dialysis membrane type and surface area, as well as shaking speed and incubation conditions have been added to ensure transparency and reproducibility of the in vitro digestion and dialysis system. In short, 1 g of each cooked sample was weighed in triplicate. Next, 95 mL of DI water was added, followed by HCl to adjust the pH to 2. The final volume was made with DI to reach 100 mL. Then, 3 mL of daily prepared pepsin solution (dissolved by 16 g of pepsin in 100 mL of 0.1 M HCl) was added before incubation. A shaking water bath (Memmert GmbH, Schwabach, Germany) was used for incubating the flasks at 37 °C for 2 h. In this study, titratable acidity was performed by adding another 20 mL of solution with fresh pepsin and pancreatin–bile extract, prepared by dissolving 0.4 g of pancreatin and 2.5 g of bile extract in 100 mL of 0.1 M NaHCO_3_, and adjusting the pH to 7.5 by different volumes of 0.5 N KOH for each sample; the final quantity of used KOH was recorded. This step was applied to neutralize sample acidity and to adjust the digestion environment to comparable conditions across all samples, thereby minimizing variability arising from differences in intrinsic acidity and ensuring consistency in the in vitro digestion process. When the incubation finished, the incubated solution was pipetted into another volumetric flask for 20 mL in triplicate. For this investigation, dialysis bags were washed by soaking in DI water for 10 min, boiling with 40% ethanol for 10 min, rinsing with DI water containing 0.01 M EDTA-Ca_2_ and 2% NaHCO_3_, rinsing again with DI water, soaking in 0.001 M NaHCO_3_, and storing at 4 °C until utilized. Using identical quantities of 0.5 N NaHCO_3_ (equal to 0.5 N KOH) in the dialysis bags, the titratable acidity of each seafood sample was assessed. A final volume of 7 mL made with DI water was added to the 50 mL volumetric vials containing a solution sample (20 mL) of pepsin digesta. The tubes were then incubated at 37 °C for 30 min in a shaking water bath. After that, a 5 mL daily prepared pancreatin–bile extract combination was added to the incubating vials, and the incubation procedure lasted for two hours. Reagent blanks were also performed using the same procedures for seafood samples in order to verify and address contamination.

ICP-QQQ-MS was finally used to measure the Se concentrations in the dialysates and residual digesta of the seafood samples. In summary, the dialysates were added with HNO_3_ in different volumes for each sample until they reached 2% *v*/*v* HNO_3_ in each sample. Then, the dialysates were filtered through a 0.22 µm nylon filter using a syringe (Merck Millipore) before operating the ICP-QQQ-MS. The residue digesta were microwave digested at the conditions listed in Supplementary Table S2 after being dried to a volume of 1 mL. Before analysis, the digested residue digesta were dried once more to a volume of 1 mL, diluted with DI water to a volume of 5 mL, and filtered through a 0.22 µm nylon filter using a syringe (Merck Millipore). Dialyzability was calculated as follows:

$$\mathrm{Dialyzability}\;(\%)\;=\;\frac{100\;\times \;dialyzed\;Se\;contents\;(µg/g\;sample)\;}{total\;Se\;contents\;(µg/g\;sample)}$$

Dialyzability referred to the proportion of the dialysate’s Se levels to the sample’s total Se content. Using ICP-QQQ-MS to measure the concentration of Se in the digested residue digesta, mass balance analysis was also carried out.

### 2.7. Accuracy and Precision

To confirm and ensure the accuracy of the Se analysis, the CRMs, NMIJ7402-a (codfish tissue with a certified value at 1.80 ± 0.20 mg/kg) and SRM1566b (oyster tissue with a certified value at 2.06 ± 0.15 mg/kg), were digested and their Se content measured in triplicate. Se concentrations measured in SRM1566b and NMIJ7402-a were 2.04 ± 0.11 mg/kg and 1.73 ± 0.06 mg/kg, respectively. There was no significant difference between the CRM’s certified and analyzed values (*p* > 0.05). The accuracy of the approach was evaluated using the percentage of relative standard deviation (%RSD). The percentage RSD of CRMs in this study, which was 3.00% for NMIJ7402-a and 5.48% for SRM1566b, demonstrated a suitable level of accuracy [27]. As a result, the Se analysis method yielded precise and accurate results.

### 2.8. This Study’s Limits of Detection (LOD) and Quantitation (LOQ)

The limit of detection (LOD) and limit of quantification (LOQ) were determined based on the standard deviation of procedural blanks (*n* = 10), using the 3σ and 10σ criteria, respectively. The LOD and LOQ were 1 µg/kg and 3 µg/kg, respectively (6.4% RSD). These values are consistent with the previously validated method [27,28,29], and units were expressed on a fresh weight basis.

### 2.9. Statistical Analysis

In addition to the Se concentrations and content in g/100 g of product, the mean standard deviation (SD) of YF, percentage of dialyzability, and other factors were calculated for boiled, fried, and grilled seafood from three sample sources. The statistical significance of the Se levels for the various seafood species, the actual retention of Se after cooking, and the percentages of dialyzability were evaluated using two-way ANOVA and Tukey’s honestly significant difference at *p* < 0.05. The experimental unit was the composite sample obtained from each market, with three independent composites collected from three different markets (*n* = 3 per species × cooking method combination). Prior to analysis, assumptions were evaluated: the normality of residuals was assessed using the Kolmogorov–Smirnov test, and the homogeneity of variances was examined using Levene’s test in SPSS. As no significant deviations were detected (*p* > 0.05), analyses were performed using untransformed data. SPSS Statistics for Windows, Version 27.0, was used to conduct the statistical analysis.

## 3. Results

### 3.1. Total Se Concentrations

Together with information on the edible portion, YF, and others, Supplementary Table S3 summarizes Se contents from selected seafood samples using different cooking methods. The greatest quantity of Se (193.9 µg/100 g product) was found in fried Indo-Pacific horseshoe crab eggs. The total Se contents of the same seafood species cooked in various ways did not alter much. Minimal Se loss during frying was demonstrated, with great true retention of Se among seafood species across various cooking techniques (72.4–100%). The chosen seafood species had YFs ranging from 0.3 to 0.8 and edible portions ranging from 19.1 to 89.1.

**Figure 1:**
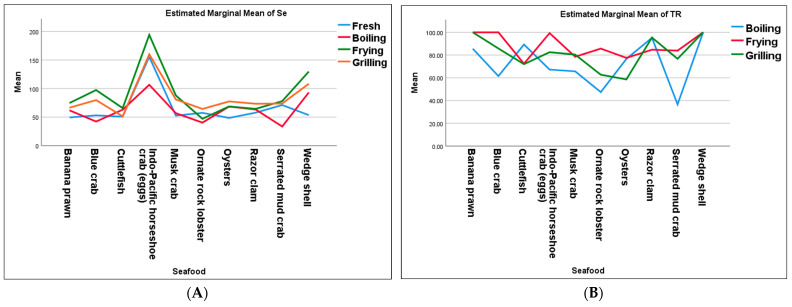
The combined impacts of various seafood species and cooking methods on Se contents; (**A**) the y-axis is µg/100 g of product and (**B**) %TR, the y-axis is the mean of the percentage.

When compared to other seafood species, the Indo-Pacific horseshoe crab (eggs) obtained the greatest concentration of Se in all cooking techniques. According to statistical test, the combined effects, or interaction effects, of seafood species and cooking techniques on Se concentration were statistically significant (*p* < 0.05) (Figure 1A). Additionally, Indo-Pacific horseshoe crab (eggs) and wedge shell had greater Se concentrations than ornate rock lobster, blue crab, and serrated mud crab (*p* < 0.05), according to data from several cooking techniques, such as boiling seafood. The greatest Se content among fried seafood was found in Indo-Pacific horseshoe crab eggs (193.9 µg/100 g of product), which differed significantly from the other seafood species (*p* < 0.05; estimated marginal means ranged from 47.1 to 129.7 µg/100 g of product) (Supplementary Table S4).

Additionally, the impact of several cooking techniques on Se concentration had a significant difference (*p* < 0.05). Fresh and boiling seafood (estimated marginal means were 65.3 and 62.9 µg/100 g of product, respectively) were significantly lower than fried and grilled seafood (estimated marginal means were 90.7 and 83.6 µg/100 g of product, respectively) (*p* < 0.05) (Table 1).

### 3.2. The Effect of Various Cooking Methods on %TR of Se

The percentage of Se retained in seafood after cooking in relation to the concentration of Se before cooking is shown by the %TR data. The combined impacts of seafood samples and cooking methods on the %TR were shown to be statistically significant (*p* < 0.05) using a two-way ANOVA with interaction and Tukey’s HSD post hoc test (Figure 1B). Boiled serrated mud crab, for instance, showed the lowest %TR (36.6%). Additionally, compared to cooked wedge shell (100%TR), boiled serrated mud crab, musk crab, ornate rock lobster, and blue crab had a considerably lower %TR of Se (*p* < 0.05). Cuttlefish had the lowest %TR (72.4%) for the frying technique, whereas wedge shell, banana prawn, and blue crab obtained 100%TR. Fried banana prawn and wedge shell (both 100%TR) showed considerably greater %TR (*p* < 0.05) than ornate rock lobster (62.7%TR) and oysters (58.6%TR), according to results from the statistical test (Supplementary Table S4). Additionally, there were significant differences in %TR between boiling, frying, and grilling (Supplementary Table S4). Compared to boiling seafood (72.5%TR), fried and grilled seafood showed significantly greater %TR (*p* < 0.05; computed means of 88.2 and 81.4%TR, respectively).

### 3.3. In Vitro Bioaccessibility of Se from Selected Seafood with Different Cooking Methods

Table 2 shows the percentages of Se bioaccessibility of the chosen seafood as measured by the in vitro equilibrium technique. For boiling seafood, the findings varied from 60.2 to 83.2%, for fried seafood, from 52.4 to 81.1%, and for grilled seafood, from 51.4 to 88.3%. According to the in vitro technique and ICP-QQQ-MS analysis, seafood produced by frying generally had slightly lower Se bioaccessibility than those prepared by boiling and grilling. Nevertheless, there were no significant differences in the proportion of Se bioaccessibility among the same seafood species whether cooked using boiling, frying, or grilling.

Of all the seafood species, boiled Indo-Pacific horseshoe crab eggs had the greatest percentage of Se bioaccessibility. Se bioaccessibility was significantly higher in boiled Indo-Pacific horseshoe crab (eggs) (83.2%) and boiled ornate rock lobster (74.1%) than in boiled musk crab (60.6%) and wedge shell (60.2%), according to a two-way ANOVA with interaction followed by Tukey’s HSD post hoc test (*p* < 0.05). For frying, Indo-Pacific horseshoe crab (eggs), banana prawn, ornate rock lobster, and serrated mud crab showed significantly higher Se bioaccessibility than blue crab, oysters, and wedge shell (*p* < 0.05). For grilling, the Indo-Pacific horseshoe crab (eggs), banana prawn, ornate rock lobster, serrated mud crab, and cuttlefish established significantly higher percentages of Se bioaccessibility than musk crab, oysters, and wedge shell (*p* < 0.05). Furthermore, boiling, frying, and grilling did not significantly alter the total percentage of Se bioaccessibility, according to the data (Table 2). Mass balance analysis was used to verify the correctness and precision of the Se equilibrium between dialyzable and residue samples. In comparison to the previously determined total Se concentration, the percentage recovery of Se was 92.8 ± 0.3 in boiled seafood, 103.8 ± 5.5 in fried seafood, and 98.2 ± 6.2 in grilled seafood (Supplementary Table S5).

## 4. Discussion

### 4.1. Edible Portion (EP) and Total Se Contents in Selected Seafood Species

The EP range is determined by the particular cooking technique used for the seafood species. Because certain internal parts, shells, and scales of seafood species are excluded before cooking in Thai households, the EP of the selected seafood in this study was different from those prior reported for Bangladesh (62–85%) [30]. In addition, a number of seafood meat components, including moisture and protein content, affect the yield factor and can cause changes in weight during cooking [31]. Furthermore, the yield factor may be related to the different fat amounts found in foods [32]. The study revealed that boiled serrated mud crab had the greatest moisture content (84 g/100 g), whereas fried blue crab had the lowest (48 g/100 g). This result agrees with the findings that fried foods lose moisture more often than boiled foods because of high-temperature heat processing, which significantly affects food moisture loss, particularly in seafood [33,34,35,36].

When compared to conventional Chinese cooking techniques, frying and other high-temperature heat processing methods had no influence on the Se degradation in Se-biofortified grains, which is consistent with the findings for total Se levels [37]. The general results of the study are similar to those for other nations, such as marine fish in Europe, where Se concentrations varied from 22 to 61 µg/100 g [11], and seafood in Japan, where they ranged from 12 to 127 µg/100 g [9]. Furthermore, the results show that Se differs from other minerals found in seafood that is often consumed in Thailand, such as vitamin D concentration, which is independent of cooking technique [27]. On the other hand, the main factor affecting Se loss after cooking could be the variety of Se forms that compose seafood. Previous studies have shown that selenocysteine and selenomethionine are the main forms of Se found in the majority of seafood species. The most prevalent form of selenium found in tuna blood is selenomethionine [38]. Because both forms of Se have a low molecular weight and dissolve easily in water, boiling causes a greater loss of Se than frying [39,40].

### 4.2. The Effects of Several Cooking Methods on %TR of Se

Shells and inedible portions had been removed from a number of the seafood species investigated during this study. Because the presence of shells and scales may reduce heat exposure during cooking, caution must be used when comparing seafood species. From this result, species-specific differences in %TR may be attributed to intrinsic characteristics such as the tissue water content, protein matrix, and Se-binding forms. Species with higher water content are more susceptible to Se loss, particularly under cooking methods involving water, due to leaching of water-soluble Se compounds [41,42]. These results of the study mostly agreed with data from the Food and Agriculture Organization of the United Nations (FAO) about the true retention of Se in seafood throughout processing, where a range of 90–100% suggested that Se is heat-resistant [43]. Furthermore, a European study that found steaming Gilthead seabream (*Sparus aurata*) had a %TR of Se of 90–100% is equivalent to the %TR findings [44].

### 4.3. In Vitro Bioaccessibility (%Dialyzability) of Se from Selected Seafood Species

According to a recent study, the high bioaccessibility of selenium in seafood species that are often consumed in Thailand is similar to that of marine fish from Europe, including swordfish, tuna, and sardine (50–83%) [18]. Also, this result is comparable to that of marine fish in Thailand (40–78%) reported in a previous study, possibly because there are no significant differences in tissue composition (i.e., protein, fat, and moisture) [20]. These results suggest that the seafood species chosen for this study have the potential to be very effective in terms of the absorption and bioavailability of Se in the human body, because the Se obtained from these seafood species is highly released from matrix tissue by human gastrointestinal simulation [45]. Therefore, dietitians should recommend some seafood species to healthy people, especially those who are susceptible to selenium insufficiency. Furthermore, a previous study found that the bioaccessibility of Se was significantly impacted by macro-elements derived from the tissues of seafood species. Dietary fiber and carbohydrate consumption had a favorable correlation with Se bioaccessibility [46]. The seafood species used in the study had no dietary fiber and a low carbohydrate content (0–1 g/100 g, data not provided). The bioaccessibility percentage of Se does not correlate with fat content, although there is a negative association between protein content and Se bioaccessibility [46]. All seafood species in the present study had protein levels of 22.6–26.7 g/100 g when boiled, 24.9–29.6 g/100 g when fried, and 24.7–26.9 g/100 when grilled (specific data not included). Additionally, their fat concentrations were 2.1–5.6 g/100 g for boiling, 6.9–13.8 g/100 g for frying, and 5.3–12.6 g/100 g for grilling. Se bioaccessibility was shown to be negatively correlated with fat (r = −0.42) and protein (r = −0.67), reported previously [20]. Furthermore, selenomethionine is the primary chemical form of selenium in seafood and the primary form of selenium in seafood dialysate, according to a prior study [46]. However, this study has not yet produced any data on Se forms.

Seafood often contains potentially harmful elements [47,48], including mercury (Hg), which is most frequently found as methyl mercury (MeHg). Se is proven to lessen mercury’s toxicity. According to reports, the marine diet’s Se:Hg molar ratio was 0.23:1 [49]. Furthermore, using the in vitro method, a previous study found that a high ratio of Se/Hg in seafood was associated with limited bioaccessibility of MeHg [50]. Lead, arsenic, and cadmium are additional potentially hazardous metals that have been shown to often contaminate marine goods [51]. Long-term use of foods tainted with these compounds may increase the risk of cancer [52]. Previous findings have determined the potentially toxic element contents and risk assessment in seafood that is frequently consumed in Thailand in order to confirm that seafood is safe to eat with contents of potentially toxic elements below the maximum levels (e.g., Pb < 0.3 mg/kg, Hg < 1.2–1.6 mg/kg, etc.) according to the FAO’s criteria [53].

Finally, Se can be found in several chemical forms, such as selenomethionine and selenocysteine, which are the most common organic forms existing in seafood and other animal products [54,55]. It has been demonstrated that inorganic forms, including selenite and selenate, are detrimental and inefficient in human bodies [56,57,58]. Further study focusing on this analysis is required to ascertain which primary Se form was acquired in the dialysate and to establish its capacity to be absorbed from these particular seafood species, as our study did not do a Se speciation analysis in the seafood dialysate.

## 5. Conclusions

The results of this present study show that the chosen seafood species are good sources of Se and may be eaten boiled, fried, or grilled. Even though heating causes some Se loss, the cooked seafood still had a high Se content. This study reports on the bioaccessibility of Se from seafood species that are commonly consumed in Thailand using different methods of cooking. For every food item under study, the bioaccessibility of Se ranges up to 88%, which is quite high. These seafood species additionally demonstrated a high Se content with in vitro bioaccessibility and after cooking. Therefore, additional studies on Se bioavailability in humans should be conducted in vivo. In conclusion, the selected ten seafood species are good sources of selenium and demonstrate relatively high bioaccessibility, suggesting that they may contribute to meeting the recommended daily intake of selenium.

## Figures and Tables

**Table 1:** The proportion of true Se retention and the estimated marginal means of Se concentration by seafood species and cooking techniques (*n* = 3).

Common Name	Estimated Marginal Means ± Standard Error	
	Se (μg/100 g of Product)	Mean of %TR
Seafood species:		
Banana prawn	63.2 ± 10.5 ^d,e^	95.2 ± 8.3 ^a^
Ornate rock lobster	52.4 ± 10.6 ^e,f,g^	65.3 ± 19.2 ^d^
Musk crab	69.6 ± 17.4 ^d,e^	74.9 ± 7.9 ^c,d^
Blue crab	68.2 ± 25.0 ^d,e^	82.5 ± 19.4 ^b,c^
Serrated mud crab	64.2 ± 20.5 ^d,e^	65.8 ± 25.4 ^d^
Cuttlefish	57.8 ± 7.7 ^e,f^	77.9 ± 9.8 ^c^
Razor clam	64.8 ± 6.4 ^d,e^	91.8 ± 6.1 ^a^
Oysters	70.3 ± 16.3 ^c,d,e^	70.9 ± 10.6 ^c,d^
Wedge shell	96.2 ± 32.1 ^b^	100.0 ± 0.0 ^a^
Indo-Pacific horseshoe crab (eggs)	154.1 ± 35.9 ^a^	83.1 ± 16.0 ^b,c^
Cooking methods for various seafood species:		
Fresh	65.3 ± 32.4 ^b^	-
Boiling	62.9 ± 22.8 ^b^	72.5 ± 20.6 ^b^
Frying	90.7 ± 42.5 ^a^	88.2 ± 10.7 ^a^
Grilling	83.6 ± 30.5 ^a^	81.4 ± 14.4 ^a^

The same letter in the same column indicates no significant difference, while different letters in the same column indicate a significant difference (*p* < 0.05) using Tukey’s HSD post hoc multiple comparisons test and two-way ANOVA. The Se content and %TR data for individual seafood shown in this table were previously reported in a previous study [13].

**Table 2:** The marginal means of %dialyzability as influenced by different seafood species and cooking methods (*n* = 3).

Common Name	Marginal Means ± Standard Error		
	Boiling (%)	Frying (%)	Grilling (%)
Seafood species:			
Banana prawn	72.4 ± 2.0 ^b^	79.0 ± 0.9 ^a^	78.1 ± 1.1 ^a,b^
Ornate rock lobster	74.1 ± 0.1 ^a,b^	79.0 ± 0.6 ^a^	83.6 ± 1.3 ^a,b^
Musk crab	60.6 ± 0.8 ^c^	66.9 ± 0.4 ^b^	63.0 ± 0.3 ^c^
Blue crab	68.3 ± 0.8 ^b,c^	62.9 ± 1.6 ^b,c^	68.7 ± 0.9 ^b,c^
Serrated mud crab	73.2 ± 0.9 ^b^	75.8 ± 0.5 ^a,b^	82.1 ± 1.5 ^a,b^
Cuttlefish	68.8 ± 0.6 ^b,c^	67.6 ± 1.6 ^b^	82.1 ± 1.5 ^a,b^
Razor clam	71.9 ± 1.0 ^b^	72.4 ± 1.5 ^a,b^	72.7 ± 2.4 ^b^
Oysters	69.9 ± 2.1 ^b,c^	58.3 ± 0.2 ^c^	52.7 ± 0.3 ^d^
Wedge shell	60.2 ± 1.4 ^c^	52.4 ± 1.7 ^c^	51.4 ± 1.0 ^d^
Indo-Pacific horseshoe crab (eggs)	83.2 ± 1.3 ^a^	81.1 ± 0.2 ^a^	88.3 ± 1.2 ^a^
Cooking methods for various seafood species:			
Average	70.2 ± 6.6 ^a^	69.5 ± 9.6 ^a^	72.0 ± 12.8 ^a^

The same letter in the same column indicates no significant difference, while different letters in the same column indicate a significant difference (*p* < 0.05) using Tukey’s HSD post hoc multiple comparisons test and two-way ANOVA (*p* < 0.05).

## Data Availability

The original contributions presented in this study are included in the article/Supplementary Materials. Further inquiries can be directed to the corresponding author.

## Supplementary Materials

The following supporting information can be downloaded at: https://www.mdpi.com/article/10.3390/foods15050873/s1, Table S1. The ten commonly consumed seafoods that were chosen for this study. Table S2. Samples digestion and total Se concentration analysis parameters. Table S3. Marginal means of moisture content, YFs, and other datas of three separate samples from each chosen seafood species (mean ± SD). Table S4. Estimated marginal means of the impacts of seafood species and cooking techniques on true Se retention and Se concentration (*n* = 3). Table S5. Mean of the mass balance recovery of each cooking method.

## Abbreviations

The following abbreviations are used in this manuscript:

ANOVA	Analysis of variance
°C	Degrees Celsius
CA	California
CRMs	Certified reference materials
DI	Deionized
EDTA-Ca_2_	Ethylenediaminetetraacetic Acid-dicalcium
EP	Edible portion
FAO	Food and Agriculture Organization
g	Gram
HCl	Hydrochloric acid
He	Helium
Hg	Mercury
HNO_3_	Nitric acid
HSD	Honestly significant difference
IBM	International Business Machines Corporation
ICP-QQQ-MS	Inductively coupled plasma triple quadrupole mass spectrometry
kDa	Kilodalton
kg	Kilogram
KOH	Potassium hydroxide
L	Liter
LOQ	Limit of quantification
LOD	Limit of detection
M	Molar
MeHG	Methyl mercury
ml	Milliliter
MWCO	Molecular Weight Cut-Off
n	Number
NaHCO_3_	Sodium bicarbonate
NMIJ	National Metrology Institute of Japan
O_2_	Oxygen
Pb	Lead
r	Correlation coefficients
RF	Radio Frequency
Rh	Rhodium
Se	Selenium
SRM	Standard Reference Material
SD	Standard deviation
SPSS	Statistical Package for the Social Sciences
TR	True retention
USA	United States of America
*v*/*v*	Volume by volume
W	Watt
YF	Yield factor
µg	Microgram
µm	Micrometer
%	Percent
