# Supplementary material for: In Vitro Bioaccessibility of Selenium in Popular Thai Seafood Across Cooking Methods
Source: Foods. 2026 Mar 4;15(5):873. doi: 10.3390/foods15050873 (PMC12984326; doi:10.3390/foods15050873)
Supplement: Supplementary file 1 [file foods-15-00873-s001.zip › Supplementary Table S1.pdf]

Supplementary Table S1. The ten commonly consumed seafoods that were chosen for this study

Common name	Scientific name	Local name	Collection (month/year)
Shrimp and prawn (captured)			
Banana prawn	*Fenneropenaeus merguiensis*	Koong Share Buay	2/2025
Ornate rock lobster	*Panulirus ornatus*	Koong Mungkorn	2/2025
Crabs (captured)			
Musk Crab	*Charybdis feriata Linnaeus*	Pu Lai Sua	2/2025
Blue crab	*Portunus pelagicus*	Pu Ma	3/2025
Serrated Mud Crab	*Scylla serrata*	Pu Dam	3/2025
Squids (captured)			
Cuttlefish	*Sepia brevimana*	Pla Muek Kradong	3/2025
Shellfish (captured)			
Razor clam	*Solen strictus Gould*	Hoi Lord	3/2025
Oysters	*Crassostrea gigas*	Hoi Nang Rom	3/2025
Wedge shell	*Mercenaria mercenaria*	Hoi Talab	3/2025
Indo-Pacific horseshoe crab (eggs)	*Tachypleus gigas*	Mangda Jan	3/2025
