# Supplementary material for: In Vitro Bioaccessibility of Selenium in Popular Thai Seafood Across Cooking Methods
Source: Foods. 2026 Mar 4;15(5):873. doi: 10.3390/foods15050873 (PMC12984326; doi:10.3390/foods15050873)
Supplement: Supplementary file 1 [file foods-15-00873-s001.zip › Supplementary Table S2.pdf]

Supplementary Table S2. Samples digestion and total Se concentration analysis parameters

Methods	Setting	
**Microwave system parameter**		
Weight of sample	0.5 g	
Begining pressure	40 bar	
Pressure	160 bar	
Step time	Step 1: 25 - 220 ºC 20 minutes<br>Step 2: 220 ºC 20 minutes	
Cooling temperature	50 ºC	
Pressure release rate	8.0 bar/min	
**ICP-QQQ-MS parameter:**	He mode	O_2 mode
Radio Frequency (RF) power	1550 W	
Sampling depth	8 mm	
Carrier gas, flow rate	1.05 L/min	
Makeup gas, flow rate	0.2 L/min	
	3 ml/min	30%
Monitor isotopes	^{77}Se, ^{78}Se, ^{82}Se, $^{78}Se^{16}O^+$, $^{80}Se^{16}O^+$, $^{82}Se^{16}O^+$	
