# Supplementary material for: In Vitro Bioaccessibility of Selenium in Popular Thai Seafood Across Cooking Methods
Source: Foods. 2026 Mar 4;15(5):873. doi: 10.3390/foods15050873 (PMC12984326; doi:10.3390/foods15050873)
Supplement: Supplementary file 1 [file foods-15-00873-s001.zip › Supplementary Table S3.pdf]

Supplementary Table S3. Marginal means of moisture content, YFs, and other datas of three separate samples from each chosen seafood species (mean ± SD)

Fish name	Sample	Edible portion (%)	YF	Moisture (g/100g)	Se concentration (μg/100g of product)	True retention of Se (%)
Banana prawn	Fresh	78.9 ± 0.9	-	81.5 ± 0.9	49.5 ± 0.3	-
	Boiled	54.9 ± 0.5	0.6 ± 0.0	71.8 ± 0.1	61.9 ± 0.4	85.6 ± 0.0
	Fried	34.5 ± 0.4	0.7 ± 0.0	68.8 ± 0.0	74.7 ± 0.8	100.0 ± 0.0
	Grilled	54.9 ± 0.5	0.7 ± 0.0	74.6 ± 0.0	66.5 ± 0.8	100.0 ± 0.0
Ornate rock lobster	Fresh	42.1 ± 0.6	-	79.4 ± 0.3	57.5 ± 0.9	-
	Boiled	38.1 ± 0.6	0.6 ± 0.0	79.8 ± 0.0	40.4 ± 0.0	47.4 ± 0.7
	Fried	35.8 ± 0.5	0.8 ± 0.0	78.0 ± 0.4	47.1 ± 0.5	85.7 ± 0.8
	Grilled	38.8 ± 0.6	0.5 ± 0.0	68.3 ± 0.3	64.3 ± 0.8	62.7 ± 0.1
Musk Crab	Fresh	30.2 ± 0.7	-	82.7 ± 0.1	52.7 ± 0.1	-
	Boiled	33.3 ± 0.6	0.6 ± 0.0	83.3 ± 0.3	56.9 ± 0.0	65.7 ± 0.9
	Fried	31.7 ± 0.6	0.4 ± 0.0	58.3 ± 0.3	87.9 ± 0.9	78.5 ± 0.5
	Grilled	33.4 ± 0.7	0.5 ± 0.0	75.9 ± 0.2	80.9 ± 0.1	80.4 ± 0.7
Blue crab	Fresh	19.1 ± 0.8	-	81.3 ± 0.5	53.1 ± 0.9	-
	Boiled	24.0 ± 0.4	0.7 ± 0.0	83.3 ± 0.3	42.3 ± 0.5	61.5 ± 0.7
	Fried	18.2 ± 0.8	0.5 ± 0.0	48.6 ± 0.4	97.4 ± 0.5	100.0 ± 0.0
	Grilled	19.6 ± 0.6	0.5 ± 0.0	66.6 ± 0.6	79.8 ± 0.9	85.8 ± 0.3
Serrated Mud Crab	Fresh	31.4 ± 0.7	-	75.2 ± 0.3	71.2 ± 0.8	-
	Boiled	33.6 ± 0.7	0.7 ± 0.0	84.2 ± 0.1	33.6 ± 0.8	36.6 ± 0.2
	Fried	30.3 ± 0.6	0.7 ± 0.0	61.3 ± 0.6	78.0 ± 0.2	83.9 ± 0.1
	Grilled	32.4 ± 0.6	0.7 ± 0.0	72.5 ± 0.0	73.8 ± 0.0	76.7 ± 0.5
Cuttlefish	Fresh	84.1 ± 0.0	-	83.5 ± 0.2	50.8 ± 0.0	-
	Boiled	89.1 ± 0.2	0.7 ± 0.0	79.6 ± 0.6	63.0 ± 0.9	89.3 ± 0.0
	Fried	82.0 ± 0.0	0.5 ± 0.0	73.0 ± 0.7	65.8 ± 0.3	72.4 ± 0.5
	Grilled	80.7 ± 0.1	0.7 ± 0.0	79.3 ± 0.6	51.5 ± 0.8	72.0 ± 0.5
Razor clam	Fresh	88.3 ± 0.9	-	78.6 ± 0.2	57.8 ± 0.0	-
	Boiled	89.1 ± 0.9	0.8 ± 0.0	73.9 ± 0.8	63.4 ± 0.5	95.2 ± 0.3
	Fried	84.6 ± 0.8	0.7 ± 0.0	67.0 ± 0.0	64.5 ± 0.4	84.7 ± 0.9
	Grilled	85.5 ± 0.9	0.7 ± 0.0	71.0 ± 0.0	73.4 ± 0.0	95.4 ± 0.7
	Fresh	68.2 ± 0.8	-	78.6 ± 0.7	48.6 ± 0.0	-
Oysters	Boiled	71.3 ± 0.7	0.5 ± 0.0	73.2 ± 0.1	68.4 ± 0.0	76.6 ± 0.3
	Fried	66.2 ± 0.6	0.4 ± 0.0	58.9 ± 0.4	86.7 ± 0.7	77.5 ± 0.4
	Grilled	67.0 ± 0.4	0.3 ± 0.0	66.2 ± 0.5	77.5 ± 0.0	58.6 ± 0.3
Wedge shell	Fresh	29.3 ± 0.6	-	77.0 ± 0.4	53.5 ± 0.2	-

Fish name	Sample	Edible portion (%)	YF	Moisture (g/100g)	Se concentration (µg/100g of product)	True retention of Se (%)
	Boiled	29.0 ± 0.6	0.6 ± 0.0	65.6 ± 0.2	93.2 ± 0.5	100.0 ± 0.0
	Fried	27.4 ± 0.5	0.5 ± 0.0	53.3 ± 0.3	129.7 ± 0.0	100.0 ± 0.0
	Grilled	28.3 ± 0.5	0.6 ± 0.0	55.5 ± 0.5	108.4 ± 0.1	100.0 ± 0.0
Indo-Pacific horseshoe crab (eggs)	Fresh	39.7 ± 0.7	-	64.1 ± 0.2	155.5 ± 0.8	-
	Boiled	47.7 ± 0.9	0.9 ± 0.0	60.6 ± 0.8	106.6 ± 0.7	67.2 ± 0.2
	Fried	38.1 ± 0.7	0.7 ± 0.0	38.9 ± 0.3	193.9 ± 0.5	99.3 ± 0.9
	Grilled	37.5 ± 0.7	0.8 ± 0.0	49.5 ± 0.9	160.1 ± 0.1	82.5 ± 0.8

The data shown in the table were part of the results reported in a previous study [13].
