# Supplementary material for: In Vitro Bioaccessibility of Selenium in Popular Thai Seafood Across Cooking Methods
Source: Foods. 2026 Mar 4;15(5):873. doi: 10.3390/foods15050873 (PMC12984326; doi:10.3390/foods15050873)
Supplement: Supplementary file 1 [file foods-15-00873-s001.zip › Supplementary Table S4.pdf]

Supplementary Table S4. Estimated marginal means of the impacts of seafood species and cooking techniques on true Se retention and Se concentration (n = 3)

Common name	Se content (µg/100g of product, Mean ± Standard Error)			True Retention of Se (%, Mean ± Standard Error)		
	**Boiling**	**Frying**	**Grilling**	**Boiling**	**Frying**	**Grilling**
Banana prawn	61.9 ± 0.40^{c,d,e,f}	74.7 ± 0.8^{d,e,f}	66.5 ± 0.8^{d,e,f}	85.6 ± 0.0^{a,b}	100.0 ± 0.0^{a}	100.0 ± 0.0^{a}
Ornate rock lobster	40.4 ± 0.0^{e,f,g}	47.1 ± 0.5^{g,h}	64.3 ± 0.8^{d,e,f}	47.4 ± 0.7^{c,d}	85.7 ± 0.8^{a,b}	62.7 ± 0.1^{b,c}
Musk Crab	56.9 ± 0.0^{c,d,e,f}	87.9 ± 0.9^{d,e,f}	80.9 ± 0.1^{b,c,d}	65.7 ± 0.9^{b,c}	78.5 ± 0.5^{a,b,c}	80.4 ± 0.7^{a,b,c}
Blue crab	42.3 ± 0.5^{e,f,g}	97.4 ± 0.5^{b,c,d}	79.8 ± 0.9^{b,c,d}	61.5 ± 0.7^{b,c}	100.0 ± 0.0^{a}	85.8 ± 0.3^{a,b,c}
Serrated Mud Crab	33.6 ± 0.8^{f,g}	78.0 ± 0.2^{d,e,f}	73.8 ± 0.0^{c,d,e}	36.6 ± 0.2^{d}	83.9 ± 0.1^{a,b}	76.7 ± 0.5^{a,b,c}
Cuttlefish	63.0 ± 0.90^{c,d,e}	65.8 ± 0.3^{e,f,g}	51.5 ± 0.8^{e,f,g}	89.3 ± 0.0^{a,b}	72.4 ± 0.5^{a,b,c,d}	72.0 ± 0.5^{a,b,c}
Razor clam	63.4 ± 0.50^{c,d,e}	64.5 ± 0.4^{e,f,g}	73.4 ± 0.0^{c,d,e}	95.2 ± 0.3^{a}	84.7 ± 0.9^{a,b}	95.4 ± 0.7^{a}
Oysters	68.4 ± 0.0^{c,d,e}	86.7 ± 0.7^{d,e,f}	77.5 ± 0.0^{c,d,e}	76.6 ± 0.3^{a,b,c}	77.5 ± 0.4^{a,b,c}	58.6 ± 0.3^{c}
Wedge shell	93.2 ± 0.5^{a,b,c}	129.7 ± 0.0^{b,c}	108.4 ± 0.1^{b,c}	100.0 ± 0.0^{a}	100.0 ± 0.0^{a}	100.0 ± 0.0^{a}
Indo-Pacific horseshoe crab [illegible]	106.6 ± 0.7^{a,b}	193.9 ± 0.5^{a}	160.1 ± 0.1^{a}	67.2 ± 0.2^{b,c}	99.3 ± 0.9^{a}	82.5 ± 0.8^{a,b,c}

The same letter in the same column indicates no significant difference, while different letters in the same column indicate significant difference ($p<0.05$), as assessed by Tukey's HSD post hoc multiple comparison test and two-way ANOVA. The data shown in the table were part of the results reported in a previous study [13].
