# Supplementary material for: In Vitro Bioaccessibility of Selenium in Popular Thai Seafood Across Cooking Methods
Source: Foods. 2026 Mar 4;15(5):873. doi: 10.3390/foods15050873 (PMC12984326; doi:10.3390/foods15050873)
Supplement: Supplementary file 1 [file foods-15-00873-s001.zip › Supplementary Table S5.pdf]

Supplementary Table S5. Mean of the mass balance recovery of each cooking method

Boiling in %recovery, mean±(SD)	Frying in %recovery, mean±(SD)	Grilling in %recovery, mean±(SD)
92.8 ± 0.3	103.8	98.2 ± 6.2
